# Abstract of the Proceedings of the Buffalo Medical Association

**Published:** 1865-01

**Authors:** Joseph A. Peters

**Affiliations:** Sec’y.


ART. III—*Abstract of the Proceedings of the Buffalo Medical Association.*

TUESDAY EVENING, December 6, 1864.

Association met pursuant to adjournment, the President, Dr. Samo, in the Chair. Present, Drs. White, Rochester, Whitney, Congar, Ring and Peters.

*Dr. White* wished to present a case of Polypus of the Uterus probably existing during pregnancy, and causing hæmorrhage after delivery. The patient was attended during her accouchment, which was attended by no untoward circumstances, by Dr. Tobie, but after a few days a hæmorrhage commenced, and when he saw her with Dr. T. three weeks after confinement she was very weak from the loss of blood. On examination he discovered a pedunculated polypus attached by its pedicle to the fundus of the uterus, which he removed by twisting off. The patient recovered, though she had a good deal of metritis, and remained anæmic for some time. He had no doubt that this polypus existed during pregnancy, which constituted the unusual feature of the case, for, though such instances are mentioned in books they are somewhat rare. Ordinarily these growths should not be removed so soon after pregnancy, on account of the danger of their giving rise to metritis, unless the danger from hæmorrhage be immediate.

*Dr. Rochester* would relate a curious instance of uterine polypus. Had been occasionally consulted by a lady about 45 years of age for neuralgia, and had proposed an examination of the uterus, but she had uniformly declined until she finally sent for him, when he found she had had repeated severe hœmorrhages, from one of which she was then suffering, accompanied by severe pain. She had been out of town and had been prescribed for, with the result of bringing on severe bearing-down pains. On examination he found a firm, hard polypus protruding from one-fourth to one-half an inch into the vagina. Did not see her again for three days, when he could detect no sign of the polypus, except that he could not pass a uterine sound into the cavity of that organ. Was he mistaken in his diagnosis? He thought not, could not doubt having seen the polypus, and no clot having been passed, nor anything resembling it, he concluded the pains had caused the polypus to protrude, and on their cessation the polypus had receded. The patient has left town, but writes that she has not had any more hæmorrhage. Would call attention to another fact. On examination he found that the remedy given by the country practitioner who saw the patient, was not ergot, as he supposed, but solution of *ferri persulphas.* Has this remedy the power of producing uterine contractions?

*Dr. Ring* remarked that the interesting case related by Prof. White, brought to mind a case that had occurred in his practice the present year, and that perhaps was worthy of mention.

Mrs. B——, aged 41 years, was taken ill early in January. She had profuse menorrhagia, continuing three or four days, then abating, and recurring about every ten days; in the intervals there was muco-sanguinolent discharge. During the month of March she was confined to her bed, much reduced in strength and flesh. At that time she was attended by a respectable practitioner in this city. Under his care the flowing somewhat abated, and she was able to sit up part of the day.

May 23d, she came under my charge. The symptoms at once excited suspicions that polypus of the uterus was the cause of her long continued sufferings. On examination per vagina, the cervix uteri was found soft, patulous, and the os enlarged. I introduced a sponge tent with the view of dilating the cavity of the uterus. On withdrawing the tent next day the dilatation was sufficient to allow of the introduction of the index finger up to the fundus. No polypus was found. Care was taken to explore the entire cavity of the uterus, using sufficient force to destroy any minute mucus polypi, that would otherwise escape destruction. On withdrawing the finger, adherent to it were found shreds, apparently the debris of what might have been polypi. Anodynes for the next day: quinia and iron, subsequently, constituted the after treatment. She has since menstruated regularly, and has regained her former good health, strength and flesh. The flowing ceased immediately after the introduction of the tent, and has not since recurred.

*Dr. White* thought diagnosis somewhat difficult in these cases, but thought the treatment referred to by Dr. Ring correct. Dilitation should always be had recourse to in cases of protracted and unaccountable hæmorrhages. Recollected a case he once saw at the Clifton House of a young lady who was nearly moribund from hæmorrhage; he plugged up the vagina with tampon, used tinct. ferri, etc., and after a few days removed the tampon, dilated the os uteri, and scarified the lining membrane with a curette. She recovered.

Had lately seen a curious case of impalement through the vagina. A woman, living on a farm, in sliding from a haymow was received on a forkstail, the tines of which were in the floor and rested the whole weight of her body on it, swinging over on it to the floor beyond. She extracted it herself and dragged herself to the door to call for help. Profuse hæmorrhage took place followed by inflammation, and in a few weeks by symptoms of suppuration, abscesses opening through the vagina and in the right iliac region, however she is now recovering. On examination the fork-handle was seen to be bloody for two inches.

*Dr. Rochester* mentioned a case of Pelvic Pyocele which he had lately seen. The patient had a tumor in the right groin and vagina, anterior to the uterus, which he proposed to open, but the lady being timid and unwilling he yielded to her solicitations to "wait a while." Subsequent propositions to open it were met in the same way, and it finally broke into the vagina, and the patient convalesced with no untoward symptoms. He spoke of it as bearing on a discussion had here some months since upon the propriety of opening such tumors through the vagina or rectum.

Also related a case of lacerated perineum following the use of instruments. The laceration extended to the sphincter ani, but did not involve that muscle. He immediately brought the edges together with four points of interrupted suture perfect union taking place.

JOSEPH A. PETERS, Sec'y.

